# Determinants and risk factors of gastroenteritis in the general population, a web-based cohort between 2014 and 2017 in France

**DOI:** 10.1186/s12889-020-09212-4

**Published:** 2020-07-21

**Authors:** Marie Ecollan, Caroline Guerrisi, Cécile Souty, Louise Rossignol, Clément Turbelin, Thomas Hanslik, Vittoria Colizza, Thierry Blanchon

**Affiliations:** 1 Sorbonne Université, INSERM, Institut Pierre Louis d’épidémiologie et de Santé Publique, IPLESP, F75012 Paris, France; 2 Department of Family Medicine, Faculté de Médecine, Université Paris Descartes, Paris, France; 3 Département de Médecine Générale, Université Paris Diderot, Paris, France; 4 Service de Médecine Interne, Hôpital Ambroise-Paré, Assistance Publique - Hôpitaux de Paris, APHP, 92100 Boulogne-Billancourt, France; 5 UFR des Sciences de la Santé Simone-Veil, Université de Versailles Saint-Quentin-en-Yvelines, 78280 Versailles, France

**Keywords:** Gastroenteritis, Epidemiology, Risk factors, Population surveillance

## Abstract

**Background:**

Although it is rarely fatal in developed countries, acute gastroenteritis (AGE) still induces significant morbidity and economic costs. The objective of this study was to identify factors associated with AGE in winter in the general population.

**Methods:**

A prospective study was performed during winter seasons from 2014 to 2015 to 2016–2017. Participants filled an inclusion survey and reported weekly data on acute symptoms. Factors associated with having at least one AGE episode per winter season were analyzed using the generalized estimating equations (GEE) approach.

**Results:**

They were 13,974 participants included in the study over the three seasons. On average, 8.1% of participants declared at least one AGE episode during a winter season. People over 60 declared fewer AGE episodes (adjusted OR (aOR) = 0.76, 95% CI [0.64; 0.89]) compared to individuals between 15 and 60 years old, as well as children between 10 and 15 (aOR = 0.60 [0.37; 0.98]). Overweight (aOR = 1.25 [1.07; 1.45]) and obese (aOR = 1.47 [1.19; 1.81]) individuals, those having frequent cold (aOR = 1.63 [1.37; 1.94]) and those with at least one chronic condition (aOR = 1.35 [1.16; 1.58]) had more AGE episodes. Living alone was associated with a higher AGE episode rate (aOR = 1.31 [1.09; 1.59]), as well as having pets at home (aOR = 1.23 [1.08; 1.41]).

**Conclusions:**

Having a better knowledge of AGE determinants will be useful to adapt public health prevention messages.

## Background

Acute gastroenteritis (AGE) is usually due to a viral infection involving the stomach and the small intestine, and its clinical picture associates diarrhoea and possible vomiting [1]. Although it is rarely fatal in developed countries, AGE still induces significant morbidity and economic costs [2, 3]. A recent study in France estimated an annual number of cases of 21,000,000, corresponding to a yearly incidence rate of 0.33 case per person [4]. Moreover 95% of individuals consulting a general practitioner for an acute diarrhoea received a drug prescription [5] and more than 80% were prescribed a sick leave [6], leading to a substantial societal cost. In a study published in 2014, results showed that stool examinations were positive for at least one enteric virus in 65% (95% CI [57–73]) of patients presenting a Winter AGE, with a predominance of noroviruses (49%) [5].

Effective preventive measures are well-known, such as hand washing education, and current prevention strategies target fragile populations at risk for complications such as elderly [7] or young children [8]. Data available to adapt the prevention strategy have been collected mainly by healthcare professionals like the French Sentinelles network [9], the English General Practice Research Framework [10] or the Continuous Morbidity Registration of the Netherlands Institute of Primary Health Care [11]. However, since AGE is a benign pathology, with only 1 patient out of 3 consulting a physician [4], using only these data is bound to cause a significant bias in the analyzed results.

Through the years, few studies have tried to assess AGE incidence and risk factors at a population level [4, 10, 12–16]. Most of them used a retrospective data collection by conducting a telephone survey of self-reported AGE in the month preceding the phone call [4, 12, 14, 16]. There have been some prospective studies on AGE conducted on the general population, but focused mainly on incidence and not on risk factors [17, 18].

The objective of this study was to characterize risk factors associated with the occurrence of AGE during winter on the general population in France. A better understanding of the profile of people having AGE among general population would help to develop more efficient and targeted public health actions.

## Methods

### Design and study population

We conducted an observational prospective study using data from the web-based GrippeNet.fr cohort [19]. GrippeNet.fr is part of a European multicentric project (Influenzanet) for syndromic surveillance during winter in the general population through online systems [20]. Participation is voluntary and anonymous after an online registration on the website. Participants are asked to fill a preliminary survey at registration regarding socio-demographic characteristics, medical history, habits and lifestyle. Then, during each winter season – called season thereafter – typically from November to April, they are invited on a weekly basis, by an email, to report and describe any symptoms on a predefined list that may have occurred in the past week. In every weekly survey, participants could select one, several or none of the symptoms suggested. The list contained the following symptoms: fever, chills, runny or blocked nose, sneezing, sore throat, cough, shortness of breath, headache, muscle/join pain, chest pain, feeling tired or exhausted (malaise), loss of appetite, colored sputum/phlegm, watery/bloodshot eyes, nausea, vomiting, diarrhoea, stomach ache, other, no symptoms. If they indicate having any symptom, they are invited to complete further information on its nature, duration or intensity. Each week, it was specified that symptoms had to be reported only if they weren’t related to any chronic condition. In case of diarrhoea, they must indicate the number of loose stool per day. The participants can report symptoms at any time and as often as they wish.

The representativeness of the GrippeNet.fr population was studied on 2011–2012 season [19]. Although it was not representative of the French general population in terms of age and gender (50–69 years old people and women being overrepresented), all age classes were represented. The GrippeNet.fr population was also found to be more frequently employed, with a higher education level of education than French population. No significant difference was found in terms of rate of studied chronic conditions, such as asthma and diabetes.

### Inclusion criteria and study period

The study was conducted over three seasons: 2014–2015 (from 29 November 2014 to 14 April 2015), 2015–2016 season (from 25 November 2015 to 8 May 2016) and 2016–2017 (from 30 November 2016 to 16 April 2017).

Any individual who filled a preliminary survey was registered as a participant for the season in the GrippeNet.fr study. In order to select only those who actively participated and provided information regularly, we included in our study participants who had filled at least one preliminary survey for the ongoing season, and reported at least three weekly surveys during a season, with at least one before, one during and one after the AGE French epidemic period. The AGE epidemic period of each season was defined by the French Sentinelles network [21]: from December the 1st, 2014 to February the 2nd, 2015 for 2014–2015 season, from January the 4th, 2016 to February the 7th, 2016 for 2015–2016 season and from November the 17th to January the 18th for 2016–2017 season. The 2016–2017 epidemic period started before the beginning of the follow-up (17 November 2016), thus, for this season, we included participants having reported at least two weekly surveys, one during and one after the epidemic period.

### Case definition

AGE episodes and their duration were identified and reconstructed using symptoms reported by the participants in the weekly surveys.

To be close to the French surveillance data, we select the definition of AGE used by the French Sentinelles network: three or more daily watery (or nearly so) stools for less than 14 days [9]. For each season, a case was defined as a participant having at least one episode of AGE, and a control as a participant having none.

We also conducted a complementary analysis with an alternative definition proposed by an expert group mandated by the WHO (the International Collaboration on Enteric Disease): three or more loose stools or any vomiting in 24 h, but excluding those due to chronic illness causing diarrhoea or vomiting, or due to drugs, alcohol or pregnancy [22].

### Participant’s characteristics

Data were collected about gender, age, household composition and residential zip code, allowing us to differentiate urban from rural participants. Questions assessed social mixing in terms of daily contact with a group of people (more than 10 individuals), with children (more than 10), with elderly (more than 10, over 65-year-old), or patients, the use of public transportation and the presence of pets at home. Data were collected on participants’ height and weight, making possible the body mass index (BMI) estimation. Smoking status was reported. Chronic conditions were evaluated by asking participants if they had taken drugs for any of the listed conditions: asthma, diabetes, heart condition, kidney condition, immunosuppression. They were also asked about the frequency of common cold or flu-like disease: never or almost never, rare (1 to 2 times a year), or often (3 or more).

### Statistical analysis

The participant’s characteristics were described by season. The same participant may have participated in one, two or three seasons. To account for this dependency, we used a logistic model using the generalized estimating equations (GEE) approach to identify variables associated with having at least one AGE episode during a season. Variables with a univariate *p-*value below 0.20 were included in the multivariable analysis. We then proceeded with a backward stepwise variables selection, using the Akaike information criterion, until reaching a final model including only variables with a *p*-value below 0.05. We conducted the same analysis with the alternate definition. All statistical analyses were performed using the R software [23] and geepack package [24].

### Ethics approval

The GrippeNet.fr study was approved by the French Advisory Committee on Information Processing in Material Research in the Field of Health (authorization 11.565) and by the French National Commission for Computing and Liberties (authorization DR-2012-024). Performing ancillary studies was mentioned in the GrippeNet.fr protocol and in the inclusion survey.

## Results

### Participation and population

During the 2014–2015 season, 3905 (59%) of the 6632 GrippeNet.fr registered individuals were considered active and were included in the study; 4906 (75%) of 6515 in 2015–2016; and 5164 (83%) of 6234 in 2016–2017 (Table 1). Overall they were 13,974 participants included in the study over the three seasons, and among them 3152 individuals were included to the study for the three seasons. During the study period, 385 participants (9.9%) declared at least one AGE in 2014–2015, 412 (8.4%) in 2015–2016 and 311 (6%) in 2016–2017. Mean number of AGE episode per participant are described in Table 1.

**Table 1:** Description of the number of participants included in the study and the number of AGE cases reported per season, using the AGE definition of the French Sentinelles network

	Season 2014–2015	Season 2015–2016	Season 2016–2017
GrippeNet.fr registered individuals^a^, n	6632	6515	6234
Active participants included in the study^b^, n (%)	3905 (59%)	4906 (75%)	5164 (83%)
Weekly surveys filled by active participants, n	75,576	100,147	82,793
AGE episode reported, n	439	476	336
Participants having at least one AGE episode, n (%)	385 (9.9%)	412 (8.4%)	311 (6.0%)
Mean number of AGE episode per participant having at least one (min – max)	1.14 (1–5)	1.16 (1–5)	1.08 (1–4)
Mean number of AGE episode per participant	0.112	0.097	0.065

^a^Any GrippeNet.fr participants having filled at least one preliminary survey

^b^GrippeNet.fr participants having filled at least three weekly surveys (one before, one during and one after the AGE epidemic period for the first two seasons, and one during and one after for the last season)

Socio-demographic characteristics, exposure and health status were similar for the three seasons (Table 2). The participants were mostly women, 60% in each season (*n* = 2326 in the first one, *n* = 2942 in the second and *n* = 3113 in the third), they lived in urban area for 81% of them (according to the season, *n* = 3145, *n* = 3973 and *n* = 4161) and were on average 53 years old in the first season, 53.6 in the second and 53 in the third. Almost half of the participants had at least one social exposure: 10 to 11% with patients, *n* = 388 in the first season, *n* = 476 the second, *n* = 555 in the third, 10% with elderly, *n* = 401, *n* = 486 and *n* = 537), 31 to 32% with a group of people, *n* = 1230, *n* = 1533 and *n* = 1677) or 24 to 33% with children, *n* = 952, *n* = 1213 and *n* = 1223). Concerning health status, 811 participants (21%) of the first season, 1079 (22%) of the second and 1098 (21%) of the third season were treated for at least one comorbidity.

**Table 2:** Socio-demographic characteristics, exposure and health characteristics of participants of the study according to the season

Variables	Season 2014–2015 n (%)	Season 2015–2016 n (%)	Season 2016–2017 n (%)
Socio-demographic characteristics			
Gender (m.d. = 0)			
Female	2326 (60%)	2942 (60%)	3113 (60%)
Male	1579 (40%)	1964 (40%)	2051 (40%)
Age (m.d. = 18)			
[0–10[	98 (3%)	188 (4%)	175 (3%)
[10–15[	104 (3%)	128 (3%)	115 (2%)
[15–60[	1970 (50%)	2541 (52%)	2660 (52%)
> 60	1732 (44%)	2042 (42%)	2205 (43%)
Household composition (m.d. =49)			
Living alone	614 (16%)	765 (16%)	838 (16%)
Living with ≥1 child	1219 (31%)	1590 (33%)	1634 (32%)
Living with adults only	2059 (53%)	2532 (52%)	2675 (52%)
Main activity (m.d. = 258)			
Working	1822 (48%)	2320 (48%)	2453 (48%)
Student	344 (9%)	487 (10%)	456 (9%)
Unemployed	109 (3%)	133 (3%)	128 (3%)
Retired	1411 (37%)	1702 (35%)	1846 (36%)
Stay at home/Sick leave	148 (4%)	171 (4%)	187 (4%)
Level of education (m.d. = 90)			
Middle school diploma	613 (16%)	707 (15%)	814 (16%)
High school diploma	696 (18%)	861 (18%)	944 (18%)
Higher education	2223 (57%)	2807 (58%)	3043 (59%)
Not concerned^a^	336 (9%)	498 (10%)	343 (7%)
Place of residency (m.d. = 0)			
Urbain	3145 (81%)	3973 (81%)	4161 (81%)
Rural	760 (19%)	933 (19%)	1003 (19%)
Exposure			
Use of public transportation (m.d. = 0)			
Yes	605 (15%)	770 (16%)	813 (16%)
No	3300 (85%)	4136 (84%)	4351 (84%)
Contacts (m.d. = 0)			
Contact with patients	388 (10%)	476 (10%)	555 (11%)
Contact with elderly	401 (10%)	486 (10%)	537 (10%)
Contact with a group of people (≥10)	1230 (31%)	1533 (31%)	1677 (32%)
Contact with children	952 (24%)	1213 (25%)	1223 (24%)
Pets at home (m.d. = 22)			
None	2127 (55%)	2711 (55%)	2851 (55%)
At least one	1774 (45%)	2187 (45%)	2303 (45%)
Health characteristics			
Common colds frequency (m.d. = 358)			
Never	1749 (45%)	2079 (44%)	2248 (44%)
Rare	1401 (36%)	1740 (37%)	1890 (37%)
Often	698 (18%)	898 (19%)	914 (18%)
Smoking status (m.d. = 16)			
Non smoker	3492 (90%)	4377 (89%)	4597 (89%)
Smoker	406 (10%)	525 (11%)	562 (11%)
Comorbidities (m.d. = 0)			
No comorbidities	3094 (79%)	3827 (78%)	4066 (79%)
At least one comorbidity^b^	811 (21%)	1079 (22%)	1098 (21%)
Treated asthma	219 (6%)	319 (7%)	303 (6%)
Treated diabetes	139 (4%)	184 (4%)	203 (4%)
Treated heart condition	397 (10%)	501 (10%)	502 (10%)
Treated kidney condition	22 (1%)	36 (1%)	29 (1%)
Treated immunosuppression	109 (3%)	144 (3%)	133 (3%)
Treated pulmonary condition	102 (3%)	139 (3%)	124 (2%)
Respiratory allergy (m.d. = 0)			
None	2609 (67%)	3267 (67%)	3378 (65%)
At least one	1296 (33%)	1639 (33%)	1786 (35%)
Pregnancy (m.d. = 67)			
Yes	39 (1%)	56 (1%)	50 (1%)
No	660 (17%)	871 (18%)	962 (19%)
Not concerned^c^	3191 (82%)	3943 (81%)	4136 (80%)
BMI (m.d. = 229)			
Underweight (< 18.5)	185 (5%)	207 (4%)	231 (5%)
Normal weight ([18.5–25[)	2208 (57%)	2791(58%)	2911 (57%)
Overweight ([25–30[)	1027 (27%)	1292 (27%)	1368 (27%)
Obese (> 30)	424 (11%)	526 (11%)	576 (11%)

*m.d.* missing data. The number correspond to the total of missing data for both seasons

^a^Children and students not having finished their studies

^b^Participants having responded they are taking medication for at least one of the condition listed below

^c^Women under 15 or above 55 and men

### Risk factor analysis

With univariable analysis (Table 3), we identify several factors associated with the risk of having at least one AGE during a winter season (*p* < 0.05): season, age, household composition, main activity, having pets, common cold or flu-like disease frequency, being treated for at least one chronic condition, having a respiratory allergy, and BMI.

**Table 3:** Factors associated with having at least one AGE episode during a winter season (univariate and multivariate analysis) among the 8811 participants-season, using the AGE definition of the French Sentinelles network

				univariate analysis		multivariate analysis	
Variable		N*	Case N (%)	OR [IC 95%]	*p*-value	OR [IC 95%]	*p*-value
Season	2014–2015	3905	385 (10%)	–	< 0.001	–	< 0.001
	2015–2016	4906	412 (8%)	0.83 [0.72–0.95]		**0.84 [0.73–0.97]**	
	2016–2017	5164	311 (6%)	0.58 [0.50–0.67]		**0.56 [0.48–0.65]**	
Sociodemographic characteristics							
Gender	Female	8381	663 (8%)	–	0.890		
	Male	5594	445 (8%)	1.01 [0.88–1.15]			
Age (years)	15 to 59	7171	617 (9%)	–	< 0.001	–	< 0.001
	< 10	461	54 (12%)	1.42 [1.04–1.93]		1.26 [0.86–1.85]	
	10 to 14	347	16 (5%)	0.52 [0.32–0.84]		**0.60 [0.37–0.98]**	
	≥ 60	5978	420 (7%)	0.80 [0.70–0.92]		**0.76 [0.64–0.89]**	
Household composition	Living with adults only	7266	536 (7%)	–	0.023	–	0.015
	Living alone	2217	208 (9%)	1.28 [1.07–1.54]		**1.31 [1.09–1.59]**	
	Living with ≥1 child	4443	361 (8%)	1.10 [0.95–1.28]		0.98 [0.82–1.17]	
Main activity	Working	6595	573 (9%)	–	0.009		
	Student	1287	91 (7%)	0.79 [0.62–1.01]			
	Unemployed	370	34 (9%)	1.07 [0.72–1.60]			
	Retired	4959	343 (7%)	0.77 [0.67–0.90]			
	Stay at home/Sick leave	506	36 (7%)	0.82 [0.56–1.20]			
Level of education	High school diploma	2564	211 (8%)	–	0.545		
	Middle school diploma	2211	157 (7%)	0.86 [0.68–1.09]			
	Higher education	7488	661 (8%)	0.99 [0.83–1.18]			
	Not concerned	961	77 (8%)	0.99 [0.74–1.31]			
Place of residency	Rural	2696	198 (7%)	–	0.269		
	Urban	11,279	910 (8%)	1.10 [0.93–1.30]			
Exposure							
Use of public transportation	No	11,787	937 (8%)	–	0.876		
	Yes	2188	171 (8%)	0.99 [0.83–1.18]			
Pets at home	None	7689	552 (7%)	–	< 0.001	–	< 0.001
	At least one	6264	555 (9%)	1.26 [1.11–1.43]		**1.23 [1.08–1.41]**	
Contact with patients	No	12,556	978 (8%)	–			
	Yes	1424	130 (9%)	1.19 [0.98–1.44]	0.083		
Contact with elderly	No	12,551	994 (8%)	–			
	Yes	1424	114 (8%)	1.00 [0.81–1.23]	0.992		
Contact with a group of people	No	9535	730 (8%)	–			
	Yes	4440	378 (9%)	1.12 [0.98–1.28]	0.092		
Contact with children	No	10,587	855 (8%)	–			
	Yes	3388	253 (7%)	0.93 [0.80–1.08]	0.325		
Health characteristics							
Common cold frequency	Never	6076	399 (7%)	–		–	< 0.001
	Rare	5031	399 (8%)	1.21 [1.04–1.40]	< 0.001	**1.17 [1.01–1.36]**	
	Often	2510	287 (11%)	1.79 [1.52–2.11]		**1.63 [1.37–1.94]**	
Smoking status	Non smoker	12,466	973 (8%)	–	0.178		
	Smoker	1493	133 (9%)	1.15 [0.94–1.41]			
Comorbidities^a^	No comorbidities	10,987	810 (7%)	–	< 0.001	–	< 0.001
	At least one comorbidity	2988	298 (10%)	1.38 [1.19–1.60]		**1.35 [1.16–1.58]**	
	Treated asthma	841	88 (10%)	1.37 [1.07–1.75]	0.012		
	Treated diabetes	526	61 (12%)	1.53 [1.10–2.12]	0.011		
	Treated heart condition	1400	133 (10%)	1.26 [1.04–1.53]	0.021		
	Treated kidney condition	87	5 (6%)	0.69 [0.29–1.64]	0.396		
	Treated immunosuppression	386	48 (12%)	1.67 [1.20–2.32]	0.023		
	Treated pulmonary condition	365	34 (9%)	1.17 [0.82–1.69]	0.414		
Respiratory allergy	None	9254	689 (7%)	–	0.008		
	At least one	4721	419 (9%)	1.20 [1.05–1.37]			
BMI	Normal weight (18.5 to 24[)	6923	560 (7%)	–	< 0.001	–	< 0.001
	Underweight (< 18.5)	1126	37 (6%)	0.84 [0.59–1.18]		0.72 [0.50–1.02]	
	Overweight (25 to 29)	3340	318 (9%)	1.23 [1.05–1.43]		**1.25 [1.07–1.45]**	
	Obese (≥ 30)	1347	171 (11%)	1.63 [1.33–1.99]		**1.47 [1.19–1.81]**	

^a^We only include the gathered variable “At least one comorbidity” in the final model, none of the individual one listed below

Regarding adjusted Odds Ratio (aOR) from the final multivariable model (Table 3), compared to individuals between 15 and 60 yo, elderly (≥ 60 yo) tend to have fewer AGE episodes (aOR = 0.76, 95% CI [0.64; 0.89]), as children between 10 and 15 y (aOR = 0.60 [0.37;0.98]). Having pets at home is associated with having AGE episode (aOR = 1.23 [1.08; 1.41]). Living alone is also associated with having AGE episode (aOR = 1.31 [1.09; 1.59]) compared to people living with adults. We also highlight three health characteristics associated with AGE: overweight (aOR = 1.25 [1.07;1.45]) and obesity (aOR = 1.47 [1.19;1.81]) compared to normal BMI, having often (aOR = 1.63 [1.37;1.94]) or rarely (aOR = 1.17 [1.01;1.36]) common cold or flu-like disease compared to never, and being treated for at least one chronic condition (aOR = 1.35 [1.16;1.58]. Participants were less likely to have AGE during the 2015/16 season (aOR = 0.84 [0.73; 0.97]) and the 2016/17 season (aOR = 0.56 [0.48; 0.65] compared to the 2014/15 season.

### Complementary analysis

Using the alternate WHO AGE definition (Table 4), 574 (14.7%) participants had at least one AGE episode in the 2014–2015 season, 682 (13.9%) in 2015–2016 and 568 (11.0%) in 2016–2017. In the final multivariable model, factors associated with at least one AGE episode per season using the previous definition were still significant (Table 4). We identified three additional risk factors: men had less AGE episodes than women (aOR = 0.81 [0.72; 0.91]), people with a lower level of education (middle school diploma) tend to have more episodes than people with high school diploma (aOR = 0.78 [0.64; 0.95]), a history of respiratory allergy was associated with AGE (aOR = 1.20 [1.07; 1.35]).

**Table 4:** Factors associated with having at least one AGE episode during a winter season (univariate and multivariate analysis) among the 8811 participants-season, using the AGE definition of the WHO expert group

				univariate analysis		multivariate analysis	
Variable		N*	Case N (%)	OR [IC 95%]	*p*-value	OR [IC 95%]	*p*-value
Season	2014–2015	3905	574 (15%)	–	< 0.001	–	< 0.001
	2015–2016	4906	682 (14%)	0.92 [0.82–1.03]		0.92 [0.82–1.04]	
	2016–2017	5164	568 (11%)	0.71 [0.63–0.80]		**0.68 [0.60–0.78]**	
Sociodemographic characteristics							
Gender	Female	8381	1168 (14%)	–	< 0.001	–	< 0.001
	Male	5594	656 (12%)	0.82 [0.74–0.92]		**0.81 [0.72–0.91]**	
Age (years)	15 to 59	7171	1018 (14%)	–		–	
	< 10	461	146 (32%)	2.82 [2.27–3.51]		**2.33 [1.35–4.02]**	
	10 to 14	347	60 (17%)	1.26 [0.93–1.71]	< 0.001	1.09 [0.60–1.95]	< 0.001
	≥ 60	5978	596 (10%)	0.67 [0.59–0.75]		**0.70 [0.61–0.80]**	
Household composition	Living with adults only	7266	833 (11%)	–		–	
	Living alone	2217	309 (14%)	1.22 [1.05–1.43]	< 0.001	**1.20 [1.03–1.41]**	0.052
	Living with ≥1 child	4443	675 (15%)	1.37 [1.22–1.53]		0.95 [0.83–1.10]	
Main activity	Working	6595	905 (14%)	–			
	Student	1287	262 (20%)	1.61 [1.37–1.89]			
	Unemployed	370	53 (14%)	1.03 [0.75–1.42]			
	Retired	4959	494 (10%)	0.69 [0.61–0.78]			
	Stay at home/Sick leave	506	56 (11%)	0.79 [0.58–1.07]	< 0.001		
Level of education	High school diploma	2564	316 (12%)	–		–	
	Middle school diploma	2211	221 (10%)	0.80 [0.66–0.97]		**0.78 [0.64–0.95]**	
	Higher education	7488	1048 (13%)	1.05 [0.91–1.22]	< 0.001	1.00 [0.86–1.16]	0.031
	Not concerned	961	228 (24%)	2.23 [1.82–2.74]		1.19 [0.71–1.99]	
Place of residency	Rural	2696	360 (13%)	–			
	Urban	11,279	1464 (13%)	0.97 [0.85–1.10]	0.605		
Exposure							
Use of public transportation	No	11,787	1450 (13%)	–			
	Yes	2188	284 (13%)	0.98 [0.85–1.13]	0.815		
Pets at home	None	7689	933 (12%)	–		–	
	At least one	6264	889 (14%)	1.20 [1.08–1.33]	< 0.001	**1.18 [1.06–1.32]**	0.009
Contact with patients	No	12,556	1603 (13%)	–			
	Yes	1424	221 (16%)	1.26 [1.08–1.48]	0.003		
Contact with elderly	No	12,551	1653 (13%)	–			
	Yes	1424	171 (12%)	0.90 [0.76–1.07]	0.226		
Contact with a group of people	No	9535	1212 (13%)	–			
	Yes	4440	612 (14%)	1.10 [0.99–1.22]	0.091		
Contact with children	No	10,587	1489 (12%)	–			
	Yes	3388	335 (16%)	1.33 [1.18–1.49]	< 0.001		
Health characteristics							
Common cold frequency	Never	6076	634 (10%)	–		–	
	Rare	5031	652 (13%)	1.24 [1.10–1.39]	< 0.001	**1.14 [1.01–1.29]**	< 0.001
	Often	2510	500 (20%)	2.05 [1.79–2.34]		**1.58 [1.37–1.82]**	
Smoking status	Non smoker	12,466	1624 (13%)	–			
	Smoker	1493	198 (13%)	1.02 [0.86–1.20]	0.860		
Comorbidities^a^	No comorbidities	10,987	1407 (13%)	–		–	
	At least one comorbidity	2988	417 (14%)	1.10 [0.97–1.25]	0.136	**1.18 [1.03–1.36]**	0.004
	Treated asthma	841	138 (16%)	1.32 [1.08–1.62]	0.008		
	Treated diabetes	526	75 (14%)	1.10 [0.83–1.46]	0.508		
	Treated heart condition	1400	172 (12%)	0.94 [0.79–1.12]	0.522		
	Treated kidney condition	87	9 (10%)	0.73 [0.33–1.59]	0.425		
	Treated immunosuppression	386	68 (18%)	1.43 [1.07–1.91]	0.017		
	Treated pulmonary condition	365	52 (14%)	1.08 [0.80–1.45]	0.633		
Respiratory allergy	None	9254	1131 (12%)	–		–	
	At least one	4721	693 (15%)	1.23 [1.10–1.37]	< 0.001	**1.20 [1.07–1.35]**	< 0.001
BMI	Normal weight (18.5 to 24[)	6923	976 (12%)	–		–	
	Underweight (< 18.5)	1126	93 (15%)	1.26 [1.00–1.60]		0.99 [0.77–1.26]	
	Overweight (25 to 29)	3340	459 (12%)	1.00 [0.88–1.14]	0.005	**1.16 [1.02–1.33]**	< 0.001
	Obese (≥ 30)	1347	240 (16%)	1.30 [1.10–1.54]		**1.35 [1.13–1.61]**	

^a^We only include the gathered variable “At least one comorbidity” in the final model, none of the individual one listed below

## Discussion

This prospective study estimated the frequency of AGE episodes during winter in the French general population and identified risk factors associated with having at least one AGE episode during this period.

Depending on the definition used and the season, between 6 and 14% of participants presented at least one AGE episode during winter. In Sweden, a study collecting health status data on a weekly basis estimated that 35% of the participants reported having at least one AGE episode in 2013 [13]. The study however adopted a more inclusive AGE definition than ours, it considered a population where young children were overrepresented, and it focused on a whole year, all factors that may explain the higher reported incidence. Other previous studies in the general population were mostly designed to evaluate a weekly or a monthly incidence rate and did not follow individuals for a long period. Therefore, they do not allow the estimation of the number of individuals having at least one AGE episode during a winter period. Our results show a decrease in the number of AGE episodes observed over the three seasons. This decrease between the 2014–2015 and 2015–2016 seasons is reflected in the national surveillance data from the French GPs’ Sentinelles network, which monitors cases of acute gastroenteritis consulting a physician during the winter period. Regarding these data, there were a slight re-increase incidence in 2016–2017, in contrast to the decrease in the number of cases observed in our study. This difference with our data is most likely due to a start of the 2015–2016 gastroenteritis epidemic before the start of the Grippenet.fr surveillance period, explaining that our study may not have accounted for AGE from the early beginning of the epidemic [25].

In our study, we identify some risk factors associated with an AGE episode, previously reported in other studies. We found that elderly people were associated to a lower risk. This association has been shown before, in France [4] and in other developed countries [12, 14, 15, 26]. However, others studies reported that infants tend to have significantly more AGE episode [4, 12, 15] than adults. We only found this association when using a more inclusive definition for AGE episodes in the complementary analysis. This can mainly be explained by the large underrepresentation of children in the GrippeNet.fr cohort [19], causing a low statistical power. In some previous studies, female gender is found to be positively associated with having an AGE episode [12, 14, 27]. We found this association when using the more inclusive definition. This association is still discussed and not systematically highlighted. Surprisingly, having possible contacts “at risk” during the day (contacts with children, with elderly, with a large group of people and with patients) were never associated with an AGE episode, whereas such associations have been found in several previous studies [5, 28, 29]. The formulation of some survey questions might be too imprecise and induced misinterpretation from participants, for instance the word “contact” is not defined at any time in the survey, as well as the duration of such contact.

Others AGE risk factors studied here have been rarely analyzed in previous work but are expected. To our knowledge, having a chronic condition has not been studied before and identified as a risk factor for AGE episode in general population. Nonetheless, some chronic conditions such as chronic kidney disease [30] or immunosuppression [31] are known to be associated with an increased risk of acute infections. This association might explain the link between overall chronic conditions and AGE episodes. Association between AGE episode and BMI status has not been found before in general population. Although this association may be partly explained by the increased frequency of functional bowel disorders among overweight people [32], participants were supposed to report only new AGE episodes and not include persistent or chronic symptoms. Overweight is already known to be associated with an increased frequency of influenza [33] and further investigation would be needed to investigate its association with AGE.

Some risk factors associated with an AGE episode in our study are less expected, like having pets at home. This risk factor has been previously studied [5, 34], but it has never been significantly associated with AGE episode. Most of the pets, such as cats, dogs, rodents or reptiles are known to carry some bacteria responsible for acute diarrhoea [35], which could partly explain this association, although the study took place during winter, when AGE are more frequently due to viral agent. The fact that individuals living alone had more episodes, was also surprising. We did not find this association in previous published literature. On the contrary, it was found that having AGE is positively associated with living with young children [4, 5, 28] or even living with more than 2 other people [4]. This discrepancy may partly be explained by the age difference: people living alone had an average age of 58 years, compared to 52 years for those living with other adults or children, (*p* < 0.001, Student-T test). Another explanation may be that people living alone have different eating habits than those living with other adults and children. A report published in 2008 by the Insee (French National Institute of Statistic and Economical Studies) showed that men living alone were more likely to buy pre-prepared dishes or to eat outdoors [36]. Eating at restaurants may increase the mixing of individuals and therefore the possible exposure to viral agents.

### Strengths and limitations

This is the first study evaluating risk factors associated with an AGE episode in France on a prospective cohort, and those factors are evaluated on a very large sample. GrippeNet.fr is an online participatory study, so it is bound to induce some bias in the representativeness of the population followed, as previously shown [19]. This particular mode of recruitment is bound to cause underrepresentation of age group with limited Internet access, such as children or elderly people, notably those living in nursing homes. These two populations may be particularly exposed to AGE epidemics, as vaccination against rotavirus is not recommended as a common practice in France among children, and community life in retirement homes is more likely to lead to outbreaks. Nevertheless, all ages, gender and level of education category are represented in the cohort, thus allowing a study on risk factor. Another important limitation to acknowledge is the design of the GrippeNet.fr cohort that was originally developed to monitor acute winter infections, including gastroenteritis, but was mainly focused on ILI-like episodes. Several potential confounding factors of AGE episodes may not have been collected in the preliminary survey, such as ages of children in household, dietary choices and exposure, or type of pet. Also, communication to potential participants was mainly oriented on ILI-like episodes, and this may have led participants to be less thorough when reporting symptoms not directly related to influenza, like diarrhoea. Nevertheless, the rate of missing data is very low, and results show that French participants contribute very regularly [37], allowing a comprehensive data collection throughout the season and minimizing the risk of undetected AGE episode.

## Conclusions

This study confirmed some well-known associations between risk-factors and having an AGE episode and found additional others. Our findings may help to target concerned population for future health information campaigns. The study also further showed how online cohorts are powerful instruments to evaluate risk factors for pathologies with a moderate rate of healthcare seeking behavior.

## Data Availability

GrippeNet.fr databases used in this study are not publicly available, in accordance with the authorization we have from the French National Commission on Informatics and Liberty (CNIL, authorization DR-2012-024). The datasets used and/or analysed during the current study are available from the authors on reasonable request.

## Abbreviations

AGE: Acute gastroenteritis
BMI: Body mass index
GEE: Generalized estimating eqs.
OR: Odds ratio
